# Interrogating cellular fate decisions with high-throughput arrays of multiplexed cellular communities

**DOI:** 10.1038/ncomms10309

**Published:** 2016-01-12

**Authors:** Sisi Chen, Andrew W. Bremer, Olivia J. Scheideler, Yun Suk Na, Michael E. Todhunter, Sonny Hsiao, Prithvi R. Bomdica, Michel M. Maharbiz, Zev J. Gartner, David V. Schaffer

**Affiliations:** 1 California Institute for Quantitative Biosciences, University of California, Berkeley, California 94720, USA; 2 Department of Bioengineering, University of California, Berkeley, California 94720, USA; 3 The UC Berkeley—UCSF Graduate Program in Bioengineering, University of California, Berkeley, California 94720, USA; 4 Department of Pharmaceutical Chemistry, University of California, San Francisco, California 94158, USA; 5 Tetrad Graduate Program, University of California, San Francisco, California 94158, USA; 6 Adheren, Emeryville, California 94662, USA; 7 Department of Electrical Engineering, University of California, Berkeley, California 94720, USA; 8 Center for Systems and Synthetic Biology, University of California, San Francisco, California 94158, USA; 9 Chemistry & Chemical Biology Graduate Program, University of California, San Francisco, California 94158, USA; 10 Department of Chemical Engineering, University of California, Berkeley, California 94720, USA; 11 Helen Wills Neuroscience Institute, University of California, Berkeley, California 94720, USA

## Abstract

Recreating heterotypic cell–cell interactions *in vitro* is key to dissecting the role of cellular communication during a variety of biological processes. This is especially relevant for stem cell niches, where neighbouring cells provide instructive inputs that govern cell fate decisions. To investigate the logic and dynamics of cell–cell signalling networks, we prepared heterotypic cell–cell interaction arrays using DNA-programmed adhesion. Our platform specifies the number and initial position of up to four distinct cell types within each array and offers tunable control over cell-contact time during long-term culture. Here, we use the platform to study the dynamics of single adult neural stem cell fate decisions in response to competing juxtacrine signals. Our results suggest a potential signalling hierarchy between Delta-like 1 and ephrin-B2 ligands, as neural stem cells adopt the Delta-like 1 phenotype of stem cell maintenance on simultaneous presentation of both signals.

## Abstract (web summary)

*In vitro* assays involving multiple cell types cannot control the stoichiometry or contact times of cell-cell interactions. Here, the authors present a patterned co-culture platform based on printed oligonucleotides capable of controlling cell-cell interactions of up to four different cell types at the single-cell level.

## Graphical abstract

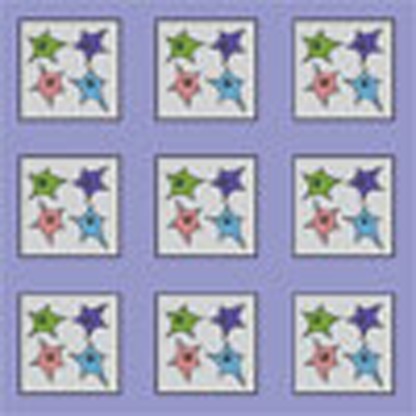

Networks of interacting cells regulate the biology and pathology of all mammalian tissues, including positive–negative selection in adaptive immune responses^1^, tumour–stromal–vascular interactions during cancer progression^2^ and stem cell-niche interactions during development and adulthood^3^. Within these intercellular signalling networks, the relative number and spatial organization of diverse cell types contributes to the behaviour of the system as a whole^4^. The capacity to reconstitute *in vitro* these networks of interacting cells, or cell communities, would offer new insights into the logic and dynamics of collective cell-decision making.

The stem cell niche is an example of a cell community containing a diversity of interacting cells that orchestrate tissue development, maintenance and repair^3^. Within this milieu, spatially restricted extracellular signals guide stem cell self-renewal and differentiation^5^. These include juxtacrine signals that require cell–cell contact, lipoprotein ligands with limited diffusion, molecules that bind proteoglycans or matrix, and soluble close-range signals^6^,^7^. For example, adult neural stem cells (NSCs)^8^,^9^,^10^ in the brain generate new neurons to modulate learning and memory, a process tightly regulated by a repertoire of neighbouring cells (astrocytes, neurons, endothelial cells and so on) that present a spectrum of signals (Eph-ephrin^11^, Notch-Delta^12^, Wnt^13^, Shh^14^ and so on). Elucidating the quantitative dynamics by which such disparate, local cues instruct sometimes mutually exclusive cell fate decisions would advance stem cell biology and regenerative medicine.

A number of methods have been developed to study networks of interacting cells. Trans-well and monolayer co-culture systems have yielded insights into intercellular signalling^13^,^15^, but in general they cannot control the stoichiometries or contact times of close-range cell–cell interactions, do not extend beyond two cell types and do not permit the longitudinal study of precisely defined groups of cells. Microfluidic and micropatterned platforms offer improved throughput and the capacity for single-cell analysis but are typically inefficient because they rely on Poisson statistics to generate arrays of interacting cells, are incapable of robust manipulation of more than two cell types at the single-cell level and restrict cell motility and proliferation^16^,^17^.

To study communication within cellular communities with improved efficiency and resolution, we engineered a high-throughput, patterned co-culture platform and investigated the effects of close-range signalling interactions on single NSC fate decisions. Our system integrates four key design criteria: (1) positional control over single cells to study their heterogeneous behaviours (single-cell resolution); (2) the capacity to simultaneously pattern multiple cell types to examine the logic of cell–cell communication within a niche (multiplexing); (3) longitudinal cell observation to reveal the dynamics of processes such as differentiation (long-term lineage tracing); and (4) robust, scalable, reproducible system performance for statistical analysis (large sample size).

With this DNA-based patterning platform, we demonstrate the unprecedented capability of reconstituting cellular communities comprised of up to four heterotypic cell types at high-throughput and with single-cell resolution. Moreover, we highlight the significantly improved efficiencies of this patterning technique over random Poisson loading as well as exhibit the strength of our system in manipulating cellular interactions by varying the initial position of patterned cell pairs, which translates to control over cell–cell contact. We then establish the promise of this platform by modelling and investigating complex cell-signalling networks. Specifically, by patterning communities of NSCs with a niche cell that expresses the Notch ligand and another that expresses the Eph ligand, this platform enables us to dissect how NSCs resolve the simultaneous presentation of competing juxtacrine signals that promote different cell fates.

## Results

### DNA-based patterning platform overview

**Figure 1:**
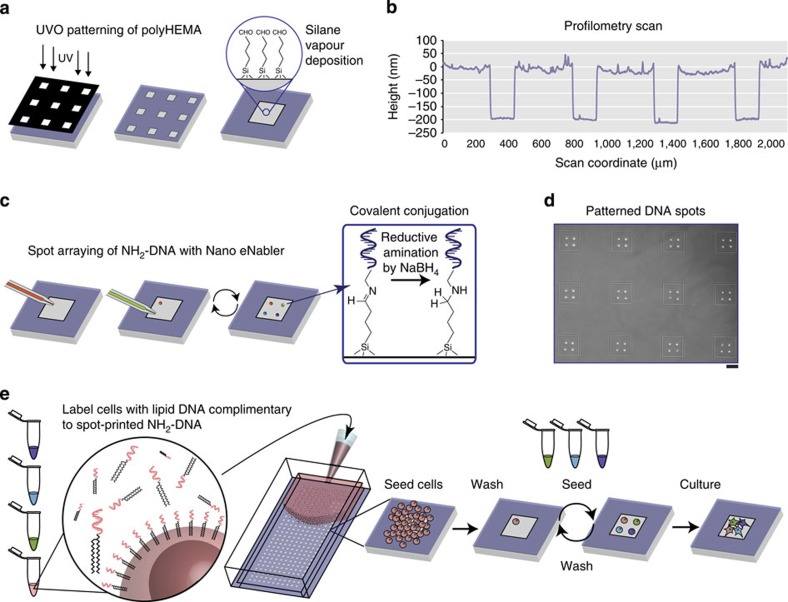
Two-step patterning process and single-cell-tethering workflow. (**a**) Microisland patterns were produced by UVO (185 nm) patterning into thin polyHEMA coatings (<0.5 μm). An aldehyde-functionalized organic silane was then vapour deposited to prepare for DNA printing. (**b**) Profilometry measurements show representative microisland features of 200 nm. (**c**) Spot arraying of NH_2_-terminated oligonucleotides within each microisland was performed using the Nano eNabler system. After arraying of single-cell-sized spots, the entire slide underwent reductive amination using NaBH_4_. (**d**) Representative image of four-component printed DNA patterns (scale bar, 100 μm). (**e**) Multiple cell populations are labelled with distinct DNA molecules presenting sequences complementary to the microisland DNA strands, washed and passed through a PDMS flow cell affixed to the patterned slides either sequentially at a density of ∼800,000 cells per cm^2^ or in mixed solutions at a density of ∼400,000 cells per cm^2^. Untethered cells are washed away, and the process is repeated for each cell type.

We fulfil the four design requirements mentioned above using a two-step patterning procedure. First, arrays of cell-adhesive ‘microislands' are generated on a non-adhesive background surface. Second, we prepare a programmably adhesive substrate by printing short oligonucleotides within each microisland, which can capture multiple cell types that present complementary DNA strands temporarily tethered to their cell membranes. The result is a geometrically organized, precisely defined community of interacting cells for biological investigation (Fig. 1).

### Fabrication of cell-adhesive microislands

In greater detail, to prepare cell-adhesive microislands, we harnessed ultraviolet–ozone (UVO) patterning to etch cell-adhesive microisland features into a non-adhesive polyhydroxyethylmethacrylate (polyHEMA) film coating an aldehyde-functionalized glass slide (Fig. 1a). Unlike other non-fouling biomaterials, polyHEMA could be deposited as a thick film and was stable for at least 7 days (Fig. 1b and Supplementary Fig. 1). The resulting array of visible microislands (Fig. 1d) obviated the need for alignment markers in subsequent printing steps, simplified image registration on consecutive days and offered a means for lineage tracing. Importantly, these microislands restricted close-range cellular signals to confined communities, yet their size could be tuned to provide space for cell migration and division as needed.

### DNA-programmed assembly for heterotypic cell patterning

To generate a programmably adhesive surface, we rely on DNA-programmed assembly^18^,^19^,^20^, a technique wherein DNA oligonucleotides are chemically incorporated into cell membranes to allow ‘velcro'-like attachment to substrates functionalized with the complementary sequences. We use direct microscale writing of DNA strands within the adhesive microislands for single-cell capture (Fig. 1c). We printed up to four orthogonal DNA sequences as cell-sized spots within each microisland (Fig. 1d), but additional sequences would enable the capture of even more cell types. After stabilization of DNA to the surface by reductive amination (Fig. 1c, box), each cell type is modified with unique lipid-conjugated complementary oligonucleotides, addressing the cell type to a specific DNA spot in the array. Cells are then serially flowed over the surface within the confines of a polydimethylsiloxane (PDMS) flow cell. Intervening washes remove unbound cells to reveal cellular communities with precisely defined composition and relative spacing (Fig. 1e).

**Figure 2:**
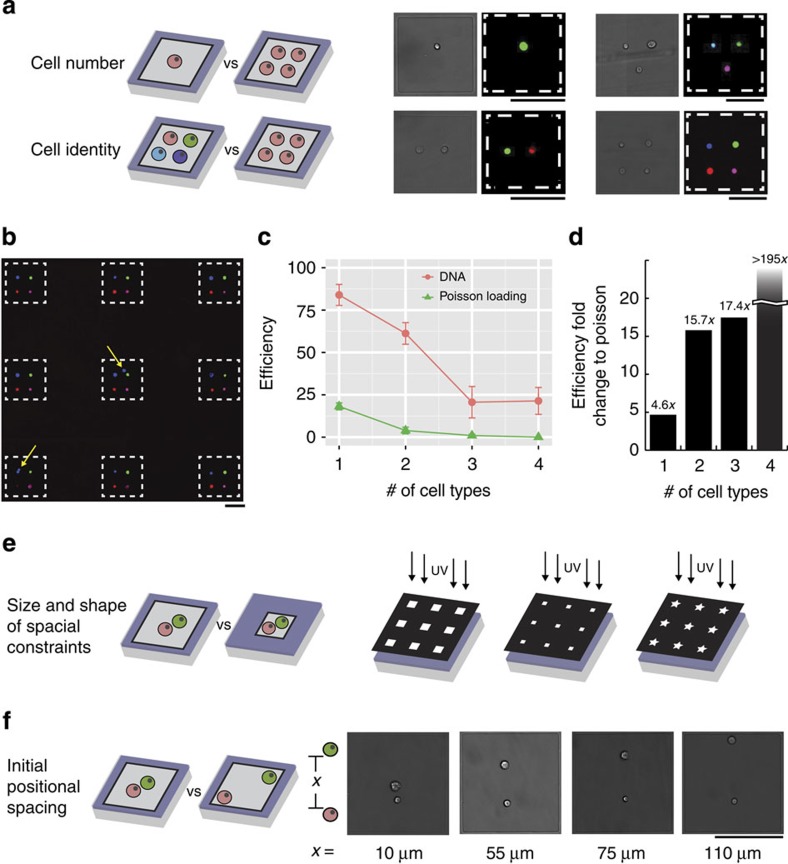
Customizable capabilities of two-step surface-patterning platform for modulating cellular interactions. (**a**) Both cell number and identity can be precisely controlled. (**b**) As an example of the latter, four MCF10A cell populations, each coloured with a different dye, were labelled with distinct DNA strands and arrayed onto microislands printed with four of the complementary DNA oligonucleotides. Seven out of the nine displayed microislands possessed the correct cellular community, with yellow arrows indicating microislands containing incorrect cellular components. (**c**) Using this DNA-based cell tethering, the efficiency of exact MCF10A cell patterning (red circles) was considerably higher than the same four cell populations plated at a low cell/surface ratio for random Poisson seeding (green triangles) of single-, double-, triple- and quadruple-cell communities. (**d**) Efficiency, or fold improvement, of our DNA-patterned compared with Poisson-loaded arrays. (**e**) Variations to the microisland features for further modulation of cell–cell communication can be achieved by changing the size and shape of the photomask used during ultraviolet (UV) etching. (**f**) DNA printing enables precise control over the initial cell positions of NSC–astrocyte (bottom cell–top cell) pairs. All error bars are s.e.m. and *n*=4. All scale bars, 100 μm.

This method for building arrays of cellular communities provides tunable control over the number, identity and initial placement of individual cells, along with the ability to define the size and shape of a community's spatial constraints (Fig. 2). For example, altering the number of DNA spots printed within a microisland determines the number of cells within each community. Moreover, printing either identical or orthogonal DNA sequences—which are highly multiplexable due to the large number of orthogonal, 20-mer oligonucleotides—dictates the capture of cells from the same or different populations (Fig. 2a). To demonstrate control over cell number and composition, we printed between one to four DNA sequences within each adhesive microisland. In parallel, we labelled four separate populations of MCF10A human mammary epithelial cells (each coloured with a different cell tracker dye) with four complementary DNA strands, which addressed each population to the corresponding DNA spot within the microisland arrays (Fig. 2a,b). Capture efficiencies were high, though occasional DNA spots neglected to capture a cell or captured more than one of the same cell type. To enable quantitative comparison to standard Poisson loading used in microfluidic and micropatterned platforms, a single population—or a mixture of two, three or all four cell populations—was seeded onto microislands lacking printed DNA at a low cell/surface area ratio (Supplementary Fig. 2). In every case, loading efficiency was higher using DNA-programmed assembly, with improvements over Poisson loading exceeding an order of magnitude for seeding with two or more cell types (Fig. 2c,d). For communities of four cell types, we achieved a nearly 25% yield compared to zero microislands seeded with the desired four cells for Poisson loading—at least a 195-fold improvement (Fig. 2d).

### Tunable control of cell–cell contact during differentiation

**Figure 3:**
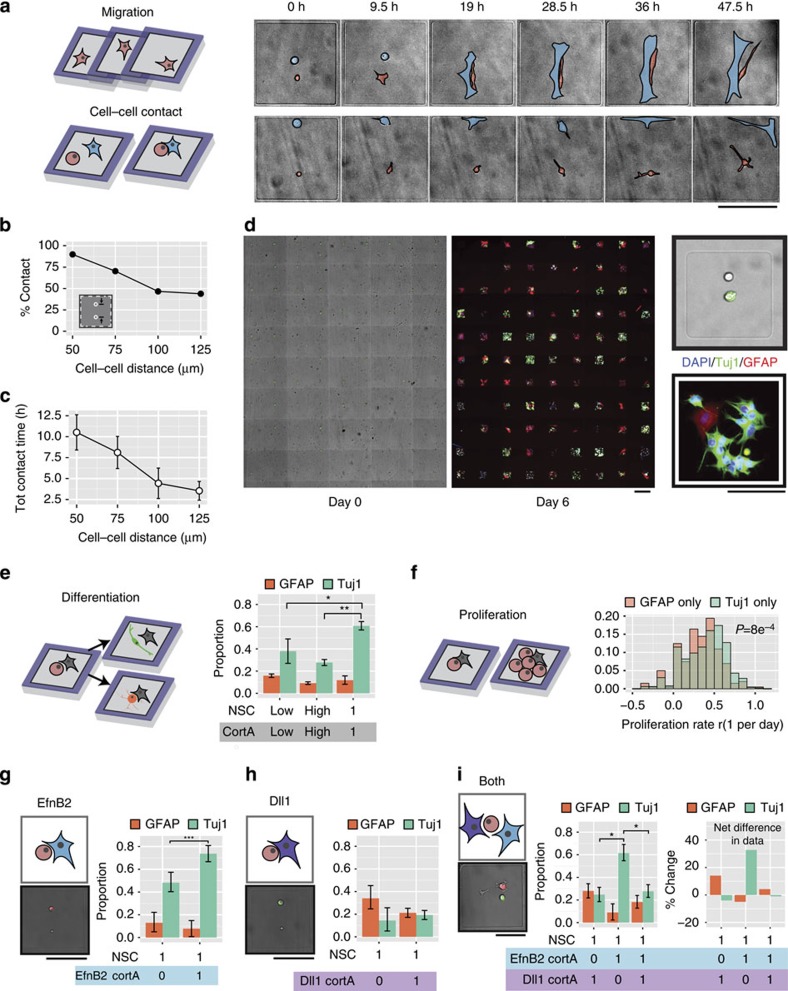
Arrays of cellular communities yield insights into cell dynamics and NSC differentiation, proliferation and signal arbitration of opposing juxtacrine signals at the single-cell level. (**a**) Migration and cell–cell contact for each microisland can be tracked with time-lapse microscopy. Representative 48-h time-lapse images illustrating the dynamics of two NSC–astrocyte pairs initially patterned at different separations. NSC highlighted in red, and astrocyte highlighted in blue. (**b**) Percent of cellular communities that showed contact increased as the initial distance separating NSC and astrocyte decreased. (**c**) Total contact times also increased as initial cell–cell distance decreased. (**d**) Cell communities could be repeatedly imaged over long timescales with subsequent visualization of differentiation markers. Representative, stitched montages of NSCs (upper) and cortical astrocytes (lower, green) immediately after patterning (left), then after immunostaining after 6 days for the neuronal marker Tuj1 and astrocyte marker GFAP (right). Higher magnification of a representative adhesive microisland shows that all progeny of this particular single NSC founder differentiated into Tuj1^+^ neurons. (**e**) NSC differentiation can be tracked for each community. When patterned with single naive astrocytes, NSCs exhibited enhanced Tuj1 differentiation and similar GFAP differentiation when compared with low-density and high-density bulk co-cultures. (**f**) Microisland confinement enabled analysis of proliferation rates. Proliferation rates (*r*) for Tuj1-biased lineages (lineages in which no GFAP cells were present) were higher than proliferation rates for GFAP-biased lineages (*P*=8e^−4^). (**g**) NSCs patterned with a single hEfnB2-overexpressing astrocyte exhibited enhanced Tuj1^+^ differentiation. (**h**) NSCs patterned with a single hDll1-overexpressing astrocyte displayed low Tuj1 expression. (**i**) When a single NSC was in the presence of both a Dll1 astrocyte and an EfnB2 astrocyte, the Dll1 phenotype (that is, reduced Tuj1) dominated. The left graph represents immunostained proportions of NSCs in each condition, and the right graph depicts immunostaining changes compared with NSCs patterned 1:1 with a naive cortical astrocyte. All error bars are 95% confidence intervals; all *P* values obtained from *t*-test. ***P<0.001, **P<0.01, *P<0.05. All scale bars, 100 μm.

In addition to controlling cell identities and numbers, altering the photomask used to etch polyHEMA during UVO patterning offers control over the size and shape of a community's spatial constraints (Fig. 2e). Moreover, the initial position of each cell within the community can be controlled by precise placement of each DNA spot within the microislands, allowing geometric arrangement of cells with programmed cell-to-cell distances (Fig. 2f). Control over these variables is important as spatial constraints determine the frequency and duration of cell–cell interactions—key determinants of cell fate decisions in the stem cell niche^21^. To examine how patterning distances regulate cell–cell contact probability and duration (Fig. 3a), we arrayed pairs of adult NSCs and primary astrocytes at intercellular distances ranging from 50 to 125 μm and conducted live imaging over 48 h. Both the percentage of cell pairs that came into contact as well as the total cell–cell contact time increased the closer the NSC–astrocyte pairs were initially patterned (Fig. 3b,c). In contrast, cells cultured without confinement in microislands—as would occur in standard co-cultures—experienced reduced interactions and often migrated away from one another (Supplementary Figs 3–5). These results demonstrate the advantage of this system to directly control cell–cell distances and confinement geometry, which lies in stark contrast to previously reported high-throughput co-culture systems that cannot control these parameters simultaneously^16^,^17^,^21^.

We next applied the platform to investigate NSC fate decisions in response to model niche cells. First, we compared NSC behaviour when co-cultured with cortical astrocytes over 6 days under two conditions: in bulk co-culture or with single astrocytes in microisland arrays (Fig. 3d and Supplementary Fig. 6). Patterns of differentiation and proliferation were quantified by recording initial and final cell counts for each microisland (Fig. 3d–f). Overall, NSCs exhibited greater neuronal differentiation (that is, expression of beta-tubulin III or Tuj1) after 6-day culture in microislands when compared with bulk co-cultures (*P*<0.05, Student's *t*-test; Fig. 3e). This observation applied to bulk co-cultures having low cell density equivalent to the overall cell density across the entire patterned substrate surface area (500 cells per cm^2^) and high cell density equivalent to cell density within each microisland (5,000 cells per cm^2^). In addition, NSCs that underwent neuronal fate commitment proliferated to a greater extent than NSCs that developed into glial fibrillary acidic protein-positive astrocytes (*P*=8e^−4^, Student's *t*-test; Fig. 3f), an interesting phenomenon also observed *in vivo*^22^.

### NSCs ‘listen' to Dll1 when presented with Dll1 and EfnB2

*In vivo*, stem cells are exposed to conflicting signals that induce mutually exclusive fate decisions. For example, Notch and Eph receptors play critical roles in mediating different cell fate decisions in the NSC niche. Notch signalling promotes the maintenance or self-renewal of early NSCs^12^,^23^, and we recently discovered that the cell surface ligand ephrin-B2 (EfnB2) presented from neighbouring astrocytes induces neuronal differentiation of NSCs^11^. As both signals are presented to NSCs in the adult niche, they likely compete to regulate stem cell fate specification—a dynamic process that our system is ideally suited to investigate at the single-cell level. Therefore, we engineered primary cortical astrocytes as model niche cells to express either EfnB2 or Delta-like 1 (Dll1) translationally coupled to a nuclear-localized fluorescent protein (Supplementary Figs 7 and 8).

We measured the distribution of cell fate decisions arising from NSCs patterned with EfnB2 astrocytes, Dll1 astrocytes or both engineered cell types. Supplementary Table 1 provides a detailed overview of the density of events that we obtained for our different community compositions (*n*=44 for 1 NSC+1 EfnB2, *n*=106 for 1 NSC+1 Dll1 and *n*=57 for 1 NSC+1 EfnB2+1 Dll1). When NSCs were cultured alone with EfnB2 astrocytes, Tuj1 expression in NSCs increased, indicating a bias towards neuronal differentiation (*P*<0.001, Student's *t*-test; Fig. 3g), and NSC proliferation rates decreased (Supplementary Fig. 9), as anticipated based on our prior work^11^. In contrast, Dll1 astrocytes biased NSCs towards low Tuj1 expression (Fig. 3h), consistent with its role in maintaining stem cell identity (Supplementary Fig. 10). In the presence of both EfnB2- and Dll1-expressing astrocytes, NSCs adopted the Dll1-responding phenotype of low Tuj1 expression (*P*<0.05, Student's *t*-test; Fig. 3i). Analogously, the distributions of percent Tuj1^+^ cells per island and the total number of Tuj1^+^ cells produced were similar when NSCs were cultured with an astrocyte expressing Dll1 alone or a Dll1 astrocyte plus an EfnB2 astrocyte (Supplementary Fig. 11b). These results suggest that, in dynamic niche microenvironments, competing juxtacrine signals from Dll1 and EfnB2 may be interpreted by NSCs as a Dll1 signal.

## Discussion

Here, we report an *in vitro* platform that tackles the shortcomings of current co-culture techniques and enables the investigation of more complex biological questions that address the role of cell–cell communication during NSC fate decisions. Using a combination of UVO and DNA-based patterning, we establish a high-throughput system for generating multiplexed arrays of cellular communities having up to four cell types. These communities can be assembled with single-cell resolution and efficiencies at least 195-fold higher than practically achievable with Poisson loading. We demonstrate robust control over community composition with regards to cell number, identity and positioning, and apply the method to study NSC behaviour and cell fate decisions in response to single and multiple signals presented from the surface of model niche cells. Our results reveal a potential signalling hierarchy between EfnB2 and Dll1 ligands during NSC differentiation.

In addition to exploring the effects of competing juxtacrine ligands, we anticipate that future applications of this technology include increasing the complexity of cellular communities by incorporating niche cell types that contribute other juxtacrine and/or paracrine signals, introducing patterned protein cues, expanding the platform to generate three-dimensional niches and quantitative real-time analysis of signalling. Together, these various approaches will yield a more complete understanding of how the logic and dynamics of intercellular signalling networks regulate the collective behaviours of cellular communities.

## Methods

### Substrate preparation

Slides were initially coated with polyHEMA to generate a non-adhesive, background surface within which adhesive features could be patterned. First, polyHEMA (Sigma) was dissolved in a sonicator for 1 h at 10 mg ml^−1^ in 100% ethanol. A volume of 150 μl of polyHEMA solution was then drop casted onto Nexterion AL (Schott) slides and allowed to dry under a clean polystyrene dish lid to block dust and slow the drying process. Slow drying over 1 h at room temperature was helpful in reducing ridges on the surface, resulting in a glossy and flat polyHEMA film. To create cell-adhesive microislands within the polyHEMA film, UVO patterning was performed using a custom quartz mask (Photosciences Inc.) and a UVO cleaner (Jelight). The quartz mask contains four 19 × 15 grids of clear square features (either 141 × 141 μm or 200 × 200 μm) arranged with a 500-μm pitch—all of which are aligned within the spatial dimensions of a Millipore 4-well EZ slide. Similar to water purification techniques that employ ultraviolet light to reduce organic contaminants, this deep ultraviolet patterning technique is thought to act through 185-nm light interacting with water and dissolved oxygen to create highly reactive hydroxyl radicals within the liquid layer, which then attack the organic polymer^24^. The very short half-life of these radicals ensure that only the clear square features are etched into the polyHEMA film. To achieve this patterning, the quartz mask was first cleaned using acetone and then irradiated in the UVO cleaner for 5 min at a distance of 5 cm to remove organic residues. A 160-μl drop of deionized water was deposited across the chrome side of the mask, and the polyHEMA-coated side of the slide was lowered onto the wetted chrome surface slowly to avoid bubble formation. Water was necessary to provide an insulating layer from the ozone generated within the UVO machine. Excess water was pressed out gently and blotted off using a lint-free TexWipe. The mask–slide assembly was then inverted onto two small stands within the machine to prevent slipping of the slide relative to the mask. This results in the polyHEMA-coated slide facing upward with the chrome mask separating the slide from the ultraviolet source, controlling for the selective passing of the ultraviolet light. The slide was then illuminated for 5 min. Exposure times of <5 min resulted in an incomplete etch (Supplementary Fig. 1b). After illumination, the slide was detached gently from the mask by flooding the surrounding area with deionized water and using tweezers to slowly pull the slide up from the mask. The slide was then rinsed with deionized water, dried under nitrogen gas and immediately placed under vacuum. With the exception of the three- and four-component experiments, all experiments employed the smaller 141 μm square size.

Because the illumination may have scavenged the organic aldehyde groups originally present on the Schott Nexterion AL slide, we reconstituted the slide with trimethoxysilane aldehyde (UCT, PSX-1050) by chemical vapour deposition in a plastic vacuum chamber under house vacuum for 1 h. Within this chamber, 100 μl of the silane was heated in a metal heat block at 110 °C. After deposition, the slide was vacuum sealed with a FoodSaver sealer and stored at room temperature until the DNA-printing step.

DNA spots of controlled sizes were printed within the adhesive microislands using a Nano eNabler system (Bioforce Nano, Ames Iowa). First, 5′-NH_2_-modified oligonucleotides were diluted to 1.5 mM in a 4 × inking buffer (20% trehalose, 0.4 mg ml^−1^ *N*-octylglucoside (pH 9.5), 900 mM NaCl and 90 μM Na Citrate). Surface-patterning tools (SPTs; BioForce Nano) of different sizes (30S and 10S versions) were cleaned by a UVO cleaner and loaded with 0.4 μl of the DNA-inking solution. 30S SPTs were used to print the 12–13-μm astrocyte-tethering spots. Spots for tethering NSCs were smaller (7–8 μm) and were printed using the 10S SPTs. These distinct, orthogonal DNA solutions were printed within close proximity of each other (10–20-μm gap). The SPTs and slides were loaded into the machine, and the humidity was allowed to equilibrate to 55–60% before printing. After DNA printing was complete, the slide was dried in a 120-°C oven for 1 min and vacuum sealed.

The printed DNA strands formed Schiff C=N bonds with the surface aldehyde. To convert the hydrolysable Schiff bases to single C–N bonds, reductive amination was performed by treatment with sodium borohydride (Sigma, 0.25% in PBS, supplemented with 0.25% LiCl) for 1 h at room temperature. Li^+^ ions were added to increase efficiency of BH_4_^−^ as a reducing agent. This step also reduced unreacted aldehyde groups on the surface to non-reactive primary alcohols. Slides were stored under vacuum at room temperature until the cell-tethering step.

### Lipid–DNA conjugates

5′-OH oligonucleotides (sequences in Supplementary Table 2) were synthesized on controlled pore glass (CPG, Glen Research) on an Applied Biosystems Expedite 8909 DNA synthesizer, as developed elsewhere. A synthetic phosphoramidite (4-Monomethoxytrityl (MMT)-Amino Modifier C6, Glen Research) was then resuspended in anhydrous acetonitrile (Fisher Scientific) according to the vendor's instructions and added to the oligonucleotides using the synthesizer. Free amine groups were generated by removing the MMT group with Deblocking Mix (Glen Research), followed by an acetonitrile wash. The CPG with oligonucleotide-amine groups was then transferred from synthesis columns to Eppendorf tubes. A C16 fatty acid (hexadecanoic acid, Sigma-Aldrich) was conjugated to oligonucleotides by adding 1 ml of a dicholoromethane (DCM, Fisher Scientific) solution containing 200 mM fatty acid, 400 mM *N*,*N*-diisopropylethylamine (Sigma-Aldrich) and 200 mM diisopropylchlorophosphoramidite (Sigma-Aldrich). Eppendorf tubes were wrapped in parafilm, secured with a cap locker and placed on a shaker overnight. The next morning, CPG beads were rinsed with a series of dicholoromethane and *N*,*N*-dimethylformamide (Sigma-Aldrich) washes and dried in a speedvac. Next, the lipid-conjugated DNA was cleaved from the CPG solid support by adding a small amount of a 1:1 mixture of ammonium hydroxide/40% methylamine (both from Sigma-Aldrich), sealing and cap-locking the tubes and incubating at 70 °C for 15–30 min. After cooling to room temperature, ammonium hydroxide/40% methylamine was evaporated overnight using a speedvac. The resulting cleaved DNA/CPG was resuspended in 700 μl of triethylamine acetic acid (Fisher Scientific) and passed through a 0.2-μm Ultrafree centrifugal filter (Millipore) to remove the CPG solid support from the cleaved DNA solution. This DNA solution was next transferred to a polypropylene vial and carried through reversed-phase high-performance liquid chromatography (HPLC) to purify the desired lipid-modified DNA product. HPLC was performed with an Agilent 1200 Series HPLC system equipped with a diode array detector monitoring at 260 and 300 nm. A C8 column (Hypersil Gold, Thermo Scientific) was used with a gradient between 8 and 95% acetonitrile over 30 min with the pure fractions collected manually at the ∼12 min mark. Fractions were lyophilized, followed by three cycles of resuspension in distilled water and further lyophilization to remove residual triethylamine acetic acid salts. Fatty acid-DNA concentrations were determined using a Thermo-Fisher NanoDrop 2000 series and measuring absorbance at 260 nm. Lipid–DNA stock solutions were resuspended at 250 μM and stored at −20 °C, with aliquots suspended in 1 × PBS to make a 5-μM working solution. CoAnchor strands were generated in similar fashion with exception to the lipid conjugation occurring on the 3′-end.

### Characterization of DNA-strand incorporation onto cells

We quantified absolute numbers of DNA strands incorporated per cell using two types of DNA: N-hydroxysuccinimide (NHS)-conjugated^25^ 20-bp oligonucleotides (purchased from Adheren, Inc.) and lipid-modified^19^ 100-bp oligonucleotides.

First, the NHS–DNA was prepared by adding 1.2 μl of activator to 175 μl of DNA solution, and the mixture was allowed to react at room temperature for 20 min. During this reaction, we detached NSCs and astrocytes, counted cells and added 2 × 10^6^ NSCs or 1 × 10^6^ astrocytes into each of three tubes. We resuspended each cell pellet with 100 μl of PBS (as a negative control), 176 μl NHS–DNA or 60 μl of lipid-DNA (5.5 mM). The NHS–DNA was reacted with cells for 20 min, and the lipid-DNA was incubated with cells for 15 min. After the reactions, the cells were diluted with 1% BSA in PBS and washed three more times. We then hybridized Alexa 488 complementary strands to the DNA-labelled cells by resuspending in 50 μl of complementary Alexa 488-conjuated DNA at 1 ng μl^−1^ and incubating on ice in the dark for 30 min. Cells were washed 3 × with 1% BSA in PBS and resuspended in a 1-ml volume before assessment on a Beckman Coulter FC 500 flow cytometer. Beads from an Alexa 488 Quantum MESF bead kit (Bang's Laboratories) were used to calibrate the total number of fluorophores conjugated to the cell surface.

Because our measurements showed that lipid-DNA was superior in the extent of DNA incorporation onto both NSCs and astrocytes (Supplementary Table 3), we used lipid-DNA for all subsequent experiments.

### Cell culture

Adult rat NSCs isolated from the hippocampi of 6-week-old female Fischer 344 rats (160–170 g)^26^ were used for stem cell signalling experiments. To promote NSC adhesion, tissue culture polystyrene plates were coated with poly-L-ornithine (Sigma) overnight at room temperature and 5 μg ml^−1^ of laminin (Invitrogen) overnight at 37 °C. Cells were cultured in monolayers in DMEM/F-12 high-glucose medium (Life Technologies) containing N-2 supplement (Life Technologies) and 20 ng ml^−1^ recombinant human FGF-2 (Peprotech), which supports self-renewal and proliferation. Medium was changed every other day, and cells were passaged using Accutase on reaching ∼80% confluency.

Rat primary cortical astrocytes from the cortices of embryonic day 19 Sprague–Dawley rats were purchased from Invitrogen (Catalogue No. N7745–100). The cells were expanded on tissue culture plates in DMEM containing 4.5 g l^−1^ glucose and 15% fetal bovine serum (FBS; Invitrogen) and initially exhibited a doubling time of ∼9 days. The cells were then adjusted to maintenance on poly-L-ornithine/laminin-coated tissue culture plates in DMEM/F-12 high-glucose containing N-2 supplement, 10% FBS and 1% penicillin/streptomycin (Gibco). Medium was changed every 2–3 days, and cells were passaged with 0.25% trypsin/EDTA as required on reaching 100% confluency.

Human mammary epithelial (MCF10A) cells were cultured in DMEM/F-12 (Invitrogen), supplemented with 5% horse serum (Invitrogen), 1% penicillin/streptomycin (Invitrogen), 0.5 μg ml^−1^ hydrocortisone (Sigma), 100 ng ml^−1^ cholera toxin (Sigma), 10 μg ml^−1^ insulin (Sigma) and 20 ng l^−1^ recombinant human epidermal growth factor (Peprotech). Similarly, medium was changed every other day, and cells were passaged with 0.25% trypsin/EDTA on reaching 80% confluency.

### hDll1 and mEfnB2 cell lines

To create astrocyte cell lines overexpressing key signalling ligands, we infected astrocytes with lentiviral vectors carrying a multicistronic cassette containing either hDelta1 (ref. 12) or hEphrinB2 (ref. 11), an nuclear localization signal (NLS)-tagged fluorophore (mCherry or Venus), and puromycin resistance (Supplementary Fig. 7). Between each coding sequence is a viral 2A peptide that self-cleaves after translation, resulting in a 1:1 stoichiometry of expression.

Plasmid DNA is transfected into HEK 293T cells in the log phase of growth, along with third-generation lentiviral helper plasmids (RSV Rev, MDL gag/pol and VSVG) using polyethylenimine at 4:1 ratio (4 μg polyethylenimine:1 μg DNA). Media is collected at 44 and 68 h after transfection, pooled, filtered with a 0.45-μm syringe filter and centrifuged in a SW28 swinging bucket rotor in a Beckman Dickinson ultracentrifuge (2 h, 24,000 r.p.m., 4 °C). A 20% sucrose layer at the bottom of each tube provides effective separation of the viral pellet from the 293T media so that the final viral suspension is free of 293T contaminants. After centrifugation, the media and the sucrose layer are aspirated, and the pellet is resuspended in sterile PBS, aliquoted and frozen at −80 °C. Infectious titres are determined by infecting astrocytes with serial dilutions of the virus, assessing infection rates by flow cytometry, and back-calculating the viral concentration using the Poisson distribution. The addition of polybrene (4 μg ml^−1^) was essential for enabling lentiviral infection for cortical astrocytes.

To generate cell lines, cortical astrocytes were infected at multiplicity of infection of 3 with 4 μg ml^−1^ polybrene. The day after infection, the media was supplemented with 10 μg ml^−1^ puromycin for 7 days through feedings and passages. For further isolation of high-expressing cells, we sorted the population by FACS using a MoFlo Cell Sorter, gating for positively fluorescent cells for both mCherry and Venus. After sorting, cells were replaced, expanded and aliquots were frozen at passages 15–18. Before each experiment, astrocytes were thawed from the same stock.

Owing to the 2A peptide linker, the NLS-XFP fluorescence could be used as a read-out of ligand expression for each cell, which we confirmed by two-colour immunoflow (Supplementary Fig. 8). To prepare cells for this analysis, astrocytes expressing NLS-mCherry hEfnB2 and NLS-mCherry hDll1 were detached from the plate using a brief accutase treatment (instead of trypsin to avoid excessive cleavage of membrane proteins). FBS-containing media was used to quench the enzymes, and 1 × 10^6^ cells were fixed using 2% paraformaldehyde and 1% BSA for 15 min. Cells were pelleted at 300*g* for 5 min and washed 2 × with PBS. Cells were blocked for 15 min in blocking buffer (5% donkey serum, 1% BSA, 0.1% Triton X-100 in PBS) and then stained with 100 μl of 1:50 rabbit polyclonal IgG for hDelta1 (sc-9102, Santa Cruz) or 1:100 rabbit polyclonal IgG for EfnB2 (HPA008999, Sigma) for 1 h on a rocking shaker at room temperature. Cells were washed 3 × with blocking buffer and then incubated in 100 μl of 1:250 Alexa 488 donkey anti-rabbit secondary antibody (Jackson Immunochemical) in the dark for 1 h on a rocking shaker at room temperature. Cells were washed 2 × in PBS and resuspended to <500 cells per μl for assessment on the Guava easyCyte 6HT. Before collecting data, fluorescence compensation was performed using 488 labelled Quantum MESF beads (Bang's Laboratories) and unstained NLS-mCherry astrocytes.

### Cell-tethering experiments

Slides were sterilized under a germicidal ultraviolet lamp in the laminar flow hood for 15 min. PDMS flow cells were plasma oxidized for 1 min (to make the surface hydrophilic) and then sealed on top of the polyHEMA patterns for each well of a four-well chamber. A non-toxic grease marker was used to line off the inlet and outlet of each flow cell to ensure that flow travels through, and not around, the flow cells. A volume of 20 μl of 2% BSA (in PBS) was added to each flow cell for 1 h to block nonspecific cell attachment.

NSCs and astrocytes were then detached and prepared at 4 × 10^6^ and 2 × 10^6^ cells, respectively, in PBS. Cells were labelled with 5 μM lipid-DNA for 10 min at room temperature and, in some cases, 5 μM of a second, CoAnchor lipid-DNA strand was successively introduced to anchor the first strand into the cell membrane (also followed by a 10-min incubation step). Following incubation, cells were washed 4 × with PBS with 3-min spins at 300*g* to pellet the cells in between washes. Cells were resuspended in 2% BSA (in PBS) to a final concentration of 4 × 10^7^ NSCs per ml or 2 × 10^7^ astrocytes per ml and stored on ice until ready for patterning. For some experiments, cell populations were combined before injecting the cell suspension (20 μl) into the flow cell. For all of the cell-settling and -washing steps, the slide was kept at 4 °C to improve strand hybridization and slow down cellular metabolism during the lengthy experimental steps.

The cells were allowed to settle to the surface for 10 min and were then cycled through the well by adding 3 μl of cell suspension at the inlet, pipetting cells up at the outlet and then adding the cell suspension back to the inlet. By cycling 15–20 × , we enhanced the probability of hybridization between matched pairs of surface-bound and cell-conjugated strands. Excess cells were washed away slowly, then vigorously, with progressively larger volumes of PBS. Gaskets from four-well Millipore EZ slides were then fastened onto the slide without removing flow cells. DMEM/F-12 with 10 μg ml^−1^ laminin was flowed through the flow cells, and the slide was incubated at 37 °C and 5% CO_2_ for 10–30 min before high-throughput imaging on the ImageXpress Micro (IXM) high-throughput automated imager. Each well was imaged in its entirety using a × 10 objective with transmitted light illumination and/or fluorescent illumination. After imaging, mixed differentiation media (50% conditioned media from NSCs in the log phase of growth, 1% FBS, 1 μM retinoic acid (Enzo Life Sciences), 1% pen/strep in DMEM/F-12 media) was added through the flow cells and used to fill the rest of the wells. Cells could then be carried through culture and, due to the transient nature of the DNA tethering (DNA linkages generally break down within hours), free to migrate and interact within their confined community over time.

### Fluorescent labelling of MCF10As for efficiency experiments

Up to four distinct MCF10A cell populations were labelled with CellTracker fluorescent dyes (Life Technologies) before cell tethering and patterning. CellTracker Green CMFDA, CellTracker Deep Red, CellTracker Violet BMQC and CellTracker Red CMPTX were prepared to a 10-mM concentration in DMSO. In all, 4 × 10^6^ MCF10As were resuspended in each CellTracker dye (0.1 μM for green, 5 μM for deep red, 10 μM for violet and 5 μM for red) in 1 × PBS for 10 min and subsequently washed 2 × with PBS. Subsequent cell-tethering steps were conducted as normal.

### Poisson loading of MCF10A populations into microislands

Up to four populations of MCF10As were labelled with distinct CellTracker fluorescent dyes for 10 min (as described in detail in the above section). Cells were then washed 2 × with 1 × PBS and prepared as a mixed population. Cells from each population were prepared at a concentration of 5 × 10^5^ cells per ml—a concentration that was previously determined by investigating a range of cell concentrations (1.25 × 10^6^ to 5 × 10^5^ cells per ml) and analysing which concentration supplied an optimal single cell per microisland coverage. A volume of 20 μl of the mixed cell population was then injected into the PDMS flow cell and allowed to settle. Slides were imaged with the IXM and quantified for efficiencies. Microislands that contained exactly one cell type from each population was considered to be efficient.

### Data analysis

Acquired images for each well (a 7 × 14 grid) were tiled to form a whole-well montage in MetaXpress. These montages were rotated with bilinear interpolation in ImageJ, scaled with consistent scalings for each set of experiments and then converted to 8-bit. Centroid coordinates for the upper left adhesive microisland were manually determined and recorded in a spreadsheet. These values were then inputted into a custom Matlab script, which cropped the images around each microisland and stored these images in an aligned array. A custom Matlab GUI, which displays the images from the array in succession, was used to record cell counts for day 0 images and immunostained images (Supplementary Fig. 12). Mean and integrated intensity values for nuclear NLS-Venus fluorescence were determined by automated segmentation using Otsu and Minimum Error thresholding, followed by end-user error correction for low-intensity cases.

Cell counts were compiled in a Matlab data structure, which was then filtered to remove sites that were uncountable (due to poor image quality, overlapping cells that make quantification impossible or imperfections in polyHEMA) and sites in which all NSCs died by day 6. The proliferation rate for each site was calculated according to the following equation:


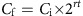


where *C*_i_ is the initial NSC count at day 0, *C*_f_ is the final count of NSC-derived progeny and *t* is the time elapsed in days. We note that this definition of proliferation includes the effects of apoptosis on cell counts. These data were then ported into R for statistical analyses and plotting using the ggplot2 package. Error bars for proportion data were generated using the MultinomialCI package on raw cell counts. A complete description of calculation metrics can be found in Supplementary Note 1.

### Immunostaining

On day 5–6 of differentiation, flow cells were removed and the cells were fixed with 4% paraformaldehyde for 10 min at room temperature. The cells were washed 3 × with PBS and then blocked in blocking buffer (PBS with 5% donkey serum and 0.3% Triton X-100) for 1 h. The cells were stained overnight at 4 °C on a rocking shaker with 1:1,000 mouse monoclonal IgG for beta-tubulin III (T8578, Sigma) and 1:1,000 rabbit polyclonal IgG for glial fibrillary acidic protein (ab7260, Abcam), diluted in blocking buffer. The next day, the antibody solution was removed, cells were washed 3 × with PBS and then incubated in the dark for 1–2 h at room temperature on a rocking shaker with secondary antibodies, 1:250 Alexa Fluor 488 donkey anti-mouse IgG (H+L) and 1:250 Cy3 donkey anti-rabbit (H+L) (all Jackson Immunochemical) in blocking buffer. After secondary incubation, cells were washed 3 × with PBS (with 1:1,000 4,6-diamidino-2-phenylindole in the second wash) and kept in PBS until imaging.

### Time-lapse experiments

NSCs and astrocytes were tethered onto DNA spots in polyHEMA-patterned and non-polyHEMA-patterned substrates, as described above. After cell tethering and washes, mixed differentiation media supplemented with 10 μg ml^−1^ laminin was flowed through the flow cells, and excess media was added to the wells. The slide was then imaged with transmitted light on the IXM using a × 10 objective at 30-min intervals for 44 h. During imaging, an environmental control chamber maintained the slide at 37 °C with a continuous supply of 5% CO_2_. Movies from 50–60 sites were collected for each of 2–3 wells in each type of substrate.

Time-lapse movies were analysed manually. For each pair of patterned cells, we recorded moments of contact and disengagement, division and death events, and the final and maximum distances between cell nuclei and membranes. The total contact time and number of contact events are calculated from these data.

### Flow cell production

Simple PDMS flow cells were produced using a 200-μm-thick mould created by stacking two white tough tags and a piece of clear tape. Using a razorblade, the sticker stack is cut to 31 × 6 mm and affixed to the bottom of a 10-cm Petri dish. Sylgard 184 (Ellsworth Adhesive) prepolymer is mixed with its curing agent at a 10:1 ratio, and 12 g of the mixture is poured onto the mould. PDMS is cured at 80 °C for 1 h, and flow cells are cut to 1 × 0.8 cm.

## Additional information

**How to cite this article:** Chen, S. *et al.* Interrogating cellular fate decisions with high-throughput arrays of multiplexed cellular communities. *Nat. Commun.* 7:10309 doi: 10.1038/ncomms10309 (2016).

## Supplementary Material

Supplementary Information Supplementary Figures 1-12, Supplementary Tables 1-3 and Supplementary Note 1
