# Supplementary material for: Interrogating cellular fate decisions with high-throughput arrays of multiplexed cellular communities
Source: Nat Commun. 2016 Jan 12;7:10309. doi: 10.1038/ncomms10309 (PMC4729920; doi:10.1038/ncomms10309)
Supplement: Supplementary Information — Supplementary Figures 1-12, Supplementary Tables 1-3 and Supplementary Note 1 [file ncomms10309-s1.pdf]

Supplementary Figures

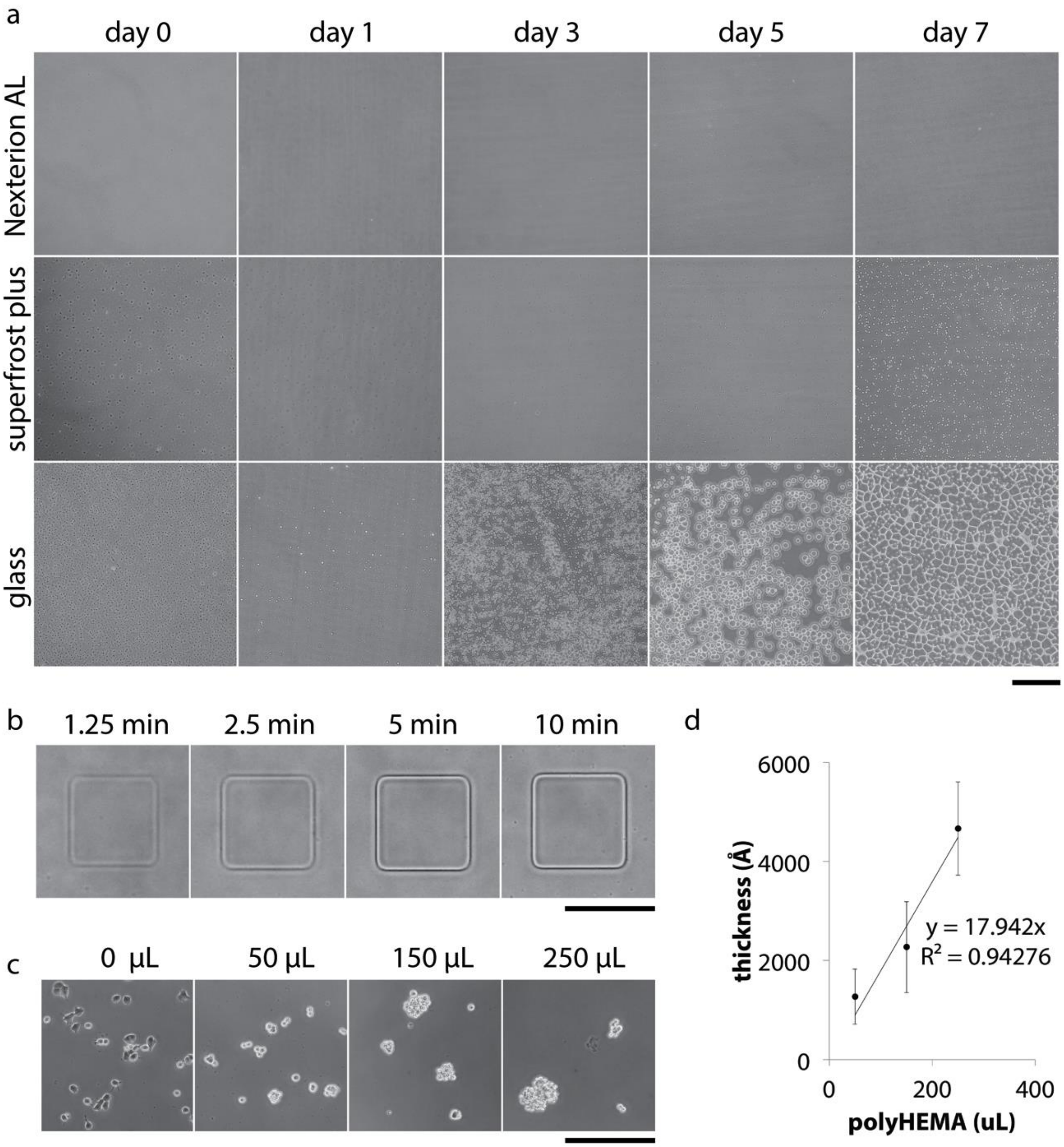

Supplementary Figure 1. PolyHEMA patterning characterization. (a) Optimizing substrate chemistry strongly enhanced polyHEMA stability in aqueous solutions. Schott Nexterion AL slides performed well relative to other options with little to no degradation over 7 days. PolyHEMA coated on plain glass slides began to detach after one day, while SuperFrost+ slides displayed slight improvement with little observable detachment until day 5. (b) Phase contrast microscopy showed that 5 minutes of deep UV illumination was sufficient to etch through our standard thickness of polyHEMA coatings (150 µL of 10 mg mL^{-1} polyHEMA). (c) PolyHEMA was effective at blocking cell adhesion down to 50 µL $slide^{-1}$. (d) The thickness of polyHEMA coating was modulated by depositing different volumes of polyHEMA solution (10 mg mL^{-1}). Profilometer measurements were performed at 9 different locations sampled over the entirety of two coated slides for each deposition. All scale bars: 200 µm.

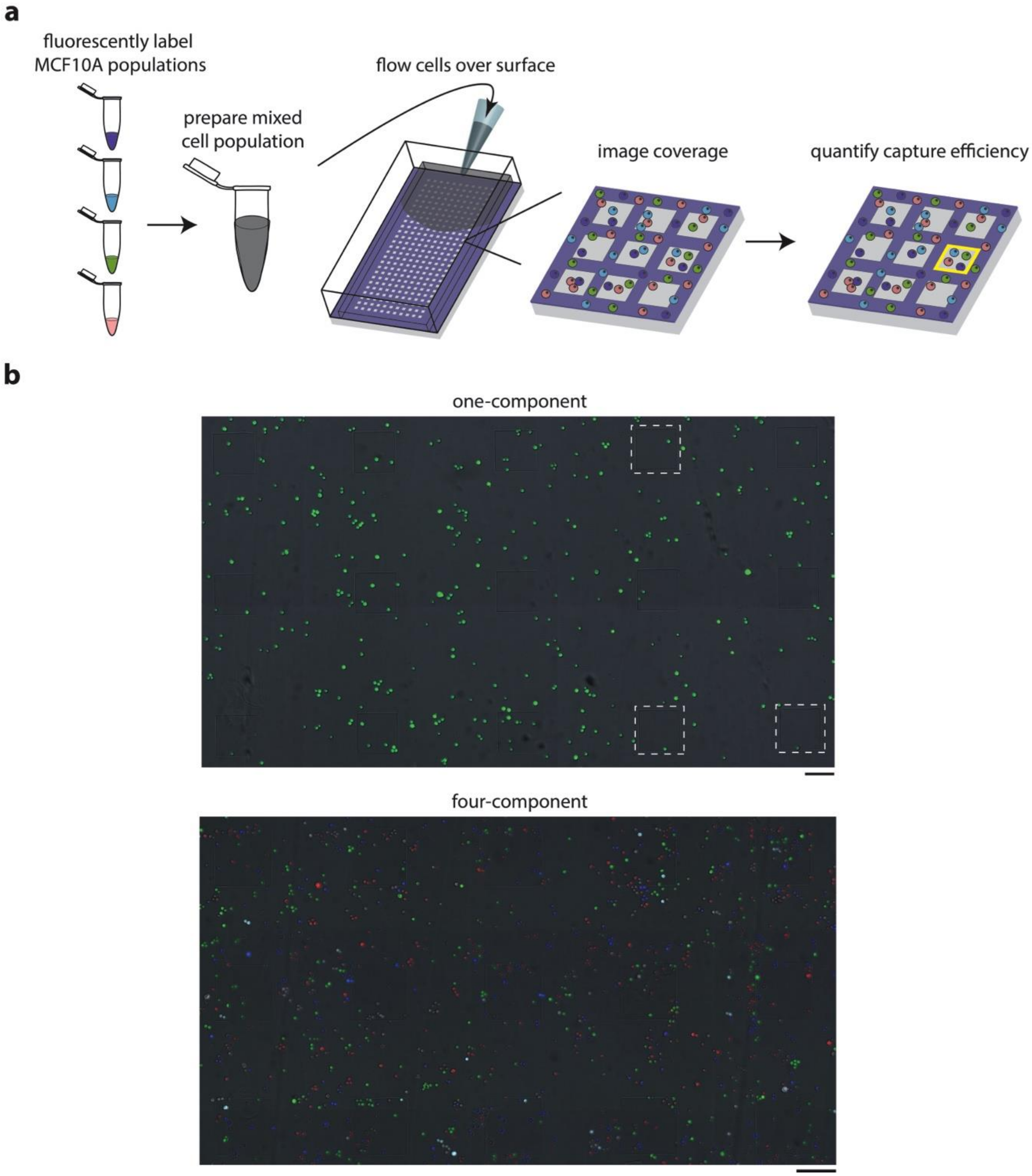

Supplementary Figure 2: Overview of experimental Poisson loading of MCF10As into microislands. (a) Schematic for conducting Poisson-loading experiment with 4 distinct, fluorescently-labeled MCF10A populations. (b) Representative one-component (top) and four-component (bottom) images of microislands that contain cells seeded using random Poisson loading. White, dashed boxes indicate microislands that possess the correct cellular components. All scale bars: 100 μm.

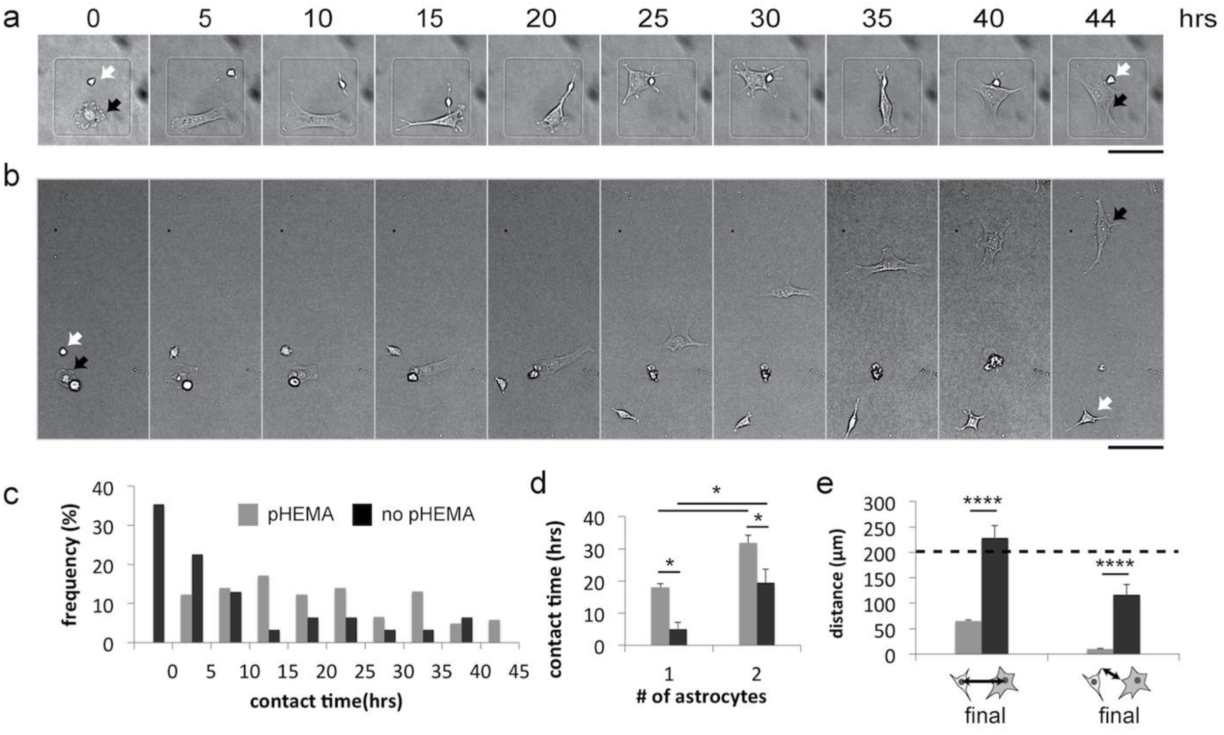

Supplementary Figure 3: Confinement to a small adhesive microisland sustains intercellular contact for extended times. (a) 141 x 141 µm adhesive microislands enabled extensive membrane contact between patterned NSCs (white arrow) and patterned astrocytes (black arrow). (b) On standard aldehyde slides lacking adhesive microislands, patterned NSCs and astrocytes migrated up to hundreds of microns away from each other over the course of 2 days. (c) Histograms of total contact times between patterned single NSCs and astrocytes showed that, without polyHEMA confinement, almost 35% of pairs made no contact at all. (d) The total amount of time that a NSC made contact with a single astrocyte was substantially increased with polyHEMA microisland confinement, and the addition of a second astrocyte further increased contact time (*: $p<0.05$, ANOVA with Tukey HSD, error bars: s.e.m). (e) The final inter-nuclear distance (left) and membrane distance (right) between patterned pairs were significantly less compared to pairs that were not confined (****: $p<1e\text{-}4$, t-test, error bars: s.e.m). The dotted line indicates the maximum corner-to-corner distance within the adhesive microislands. All scale bars: 100 µm.

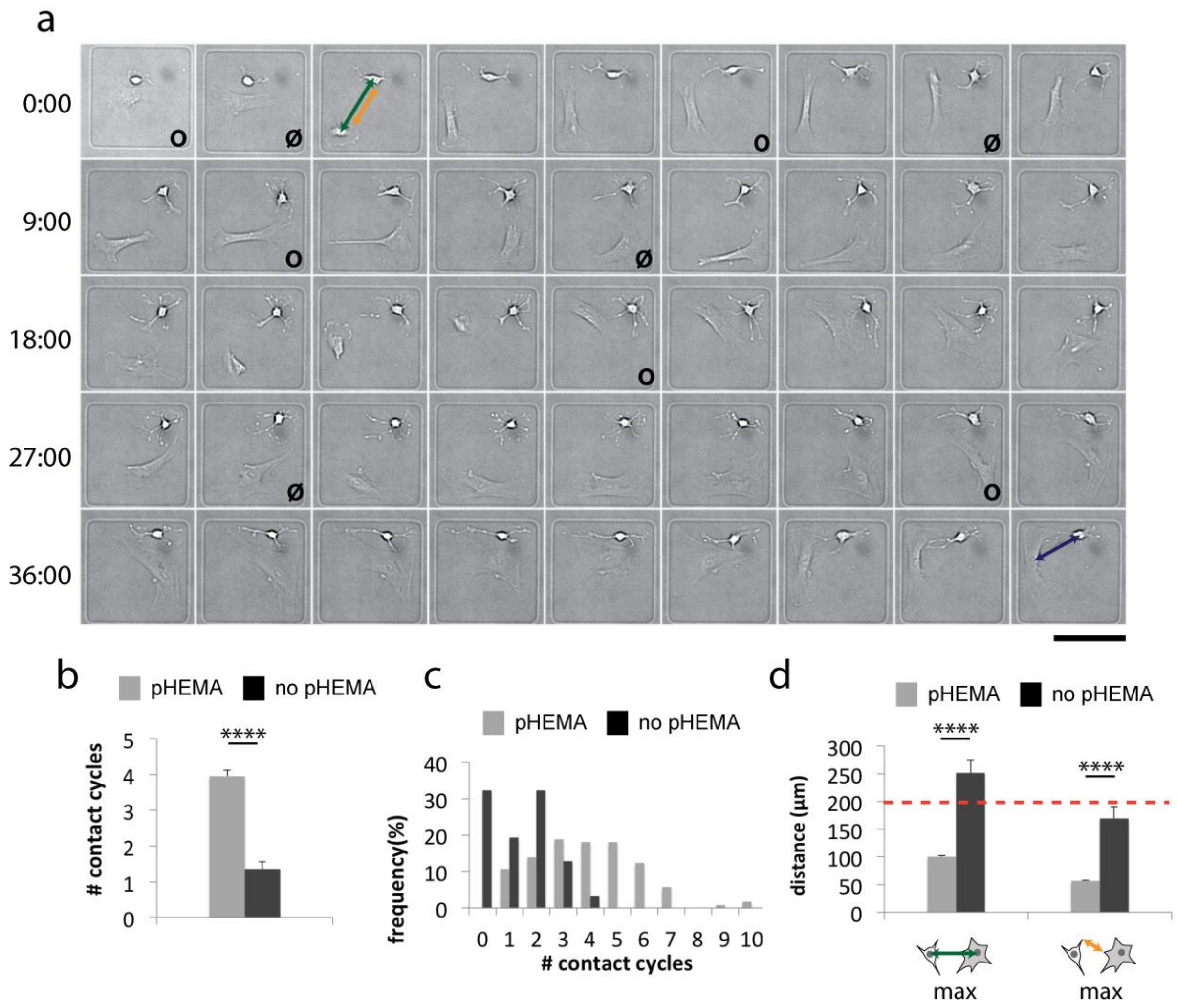

Supplementary Figure 4. Additional timelapse montages to show analysis framework. (a) DNA-tethered NSC and astrocyte pairs in polyHEMA patterns make several apparent cycles of contact (O) and disengagement (Ø). The green arrow shows the maximum distance between nuclei, and the yellow arrow shows the maximum cell membrane distance over the course of the experiment. The blue arrow shows the final distance between nuclei at 44 hours and, in this case, the final cell distance was 0 since the membranes were in contact at 44 hours. Hours are denoted on the left. (b) PolyHEMA-bounded pairs experienced significantly more contact cycles over 44 hours compared to pairs without polyHEMA (where representative images for no polyHEMA are shown in Supplementary Figure 5) ($p<1e-13$, error bars: s.e.m.). (c) The histogram of contact cycles shows a wider spread for polyHEMA-bounded pairs. (d) The maximum nuclear and membrane distances (green and yellow) between pairs were also significantly lower for polyHEMA-bounded pairs ($p<1e-5$, error bars: s.e.m.). All scale bars: 100 µm.

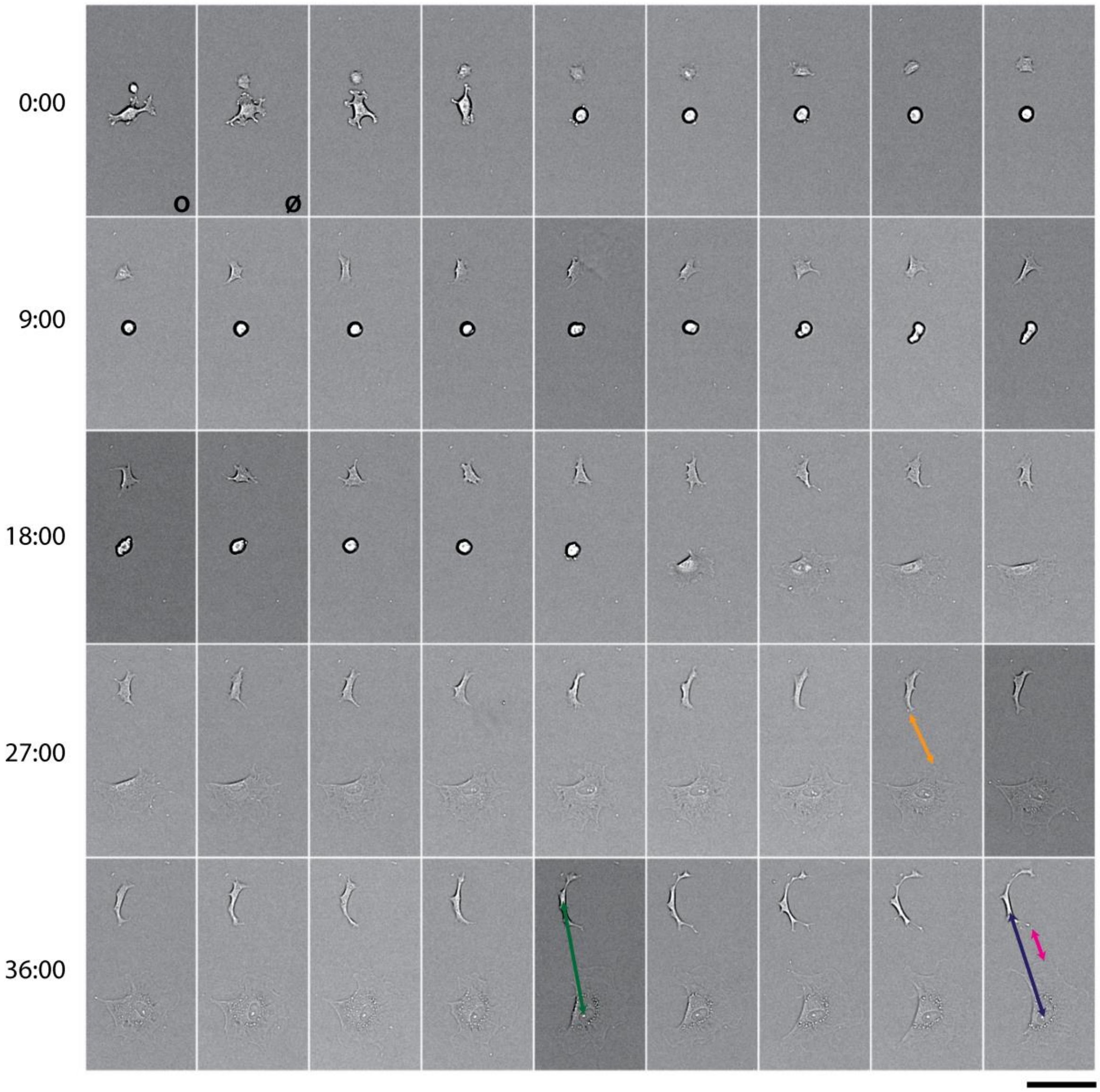

Supplementary Figure 5: Additional timelapse montages of cells patterned without polyHEMA boundaries. Notated using the same strategy as described in Supplementary Figure 4. The magenta arrow shows the final cell membrane distance.

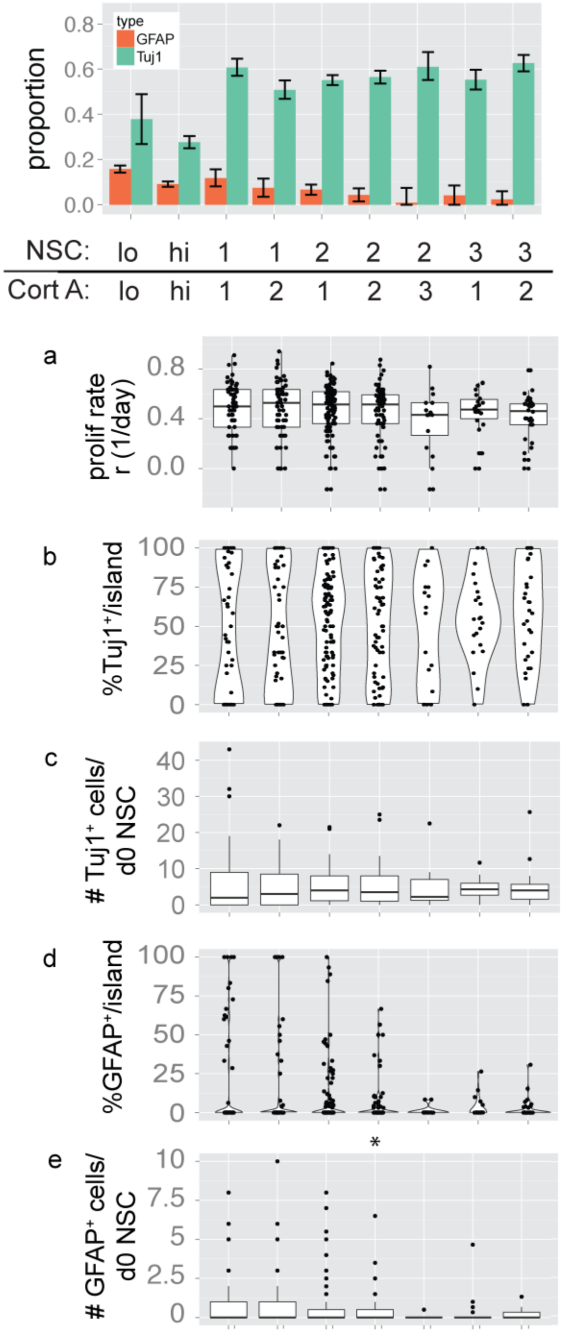

Supplementary Figure 6. Additional plots for NSCs patterned with naïve astrocytes. NSCs patterned with 1, 2, and 3 astrocytes exhibited higher differentiation rates than NSCs patterned in bulk, at either high (5000 cells cm^{-2}) or low density (500 cells cm^{-2}). (a) Proliferation rates. (b) Distribution of % $Tuj1^{+}$ $island^{-1}$ shows that increasing the number of initial NSC progenitors cause wider bellies in the distributions, reflecting the lower likelihood that all progeny adopt the same fate. (c) Boxplot of the number of $Tuj1^{+}$ cells generated from each initial NSC. (d) Violin plots of the distributions of % $GFAP^{+}$ $island^{-1}$. (e) Boxplot of the number of $GFAP^{+}$ cells (initial NSC)$^{-1}$. All error bars are 95% confidence intervals.

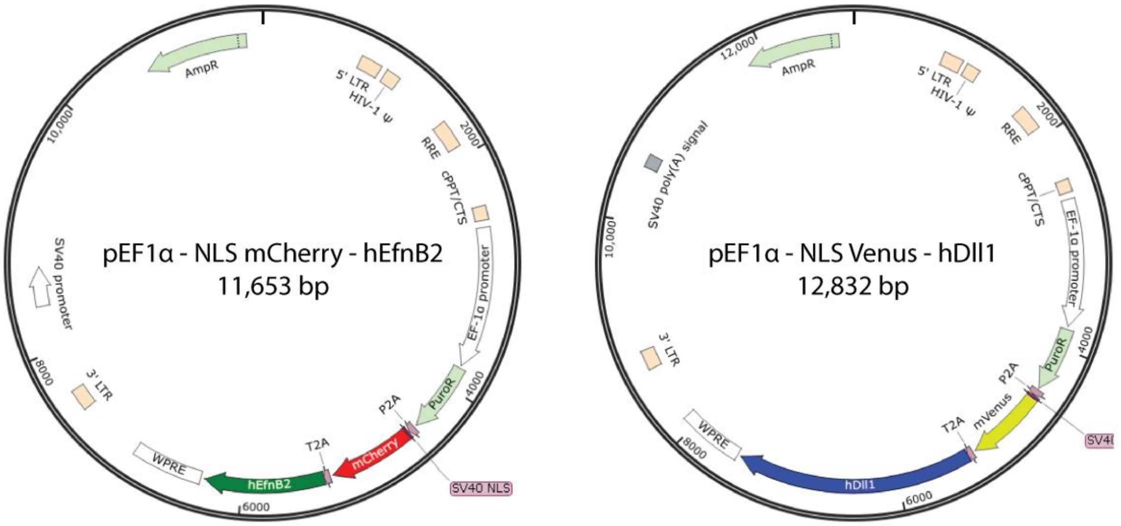

Supplementary Figure 7. Plasmid maps for expression vectors.

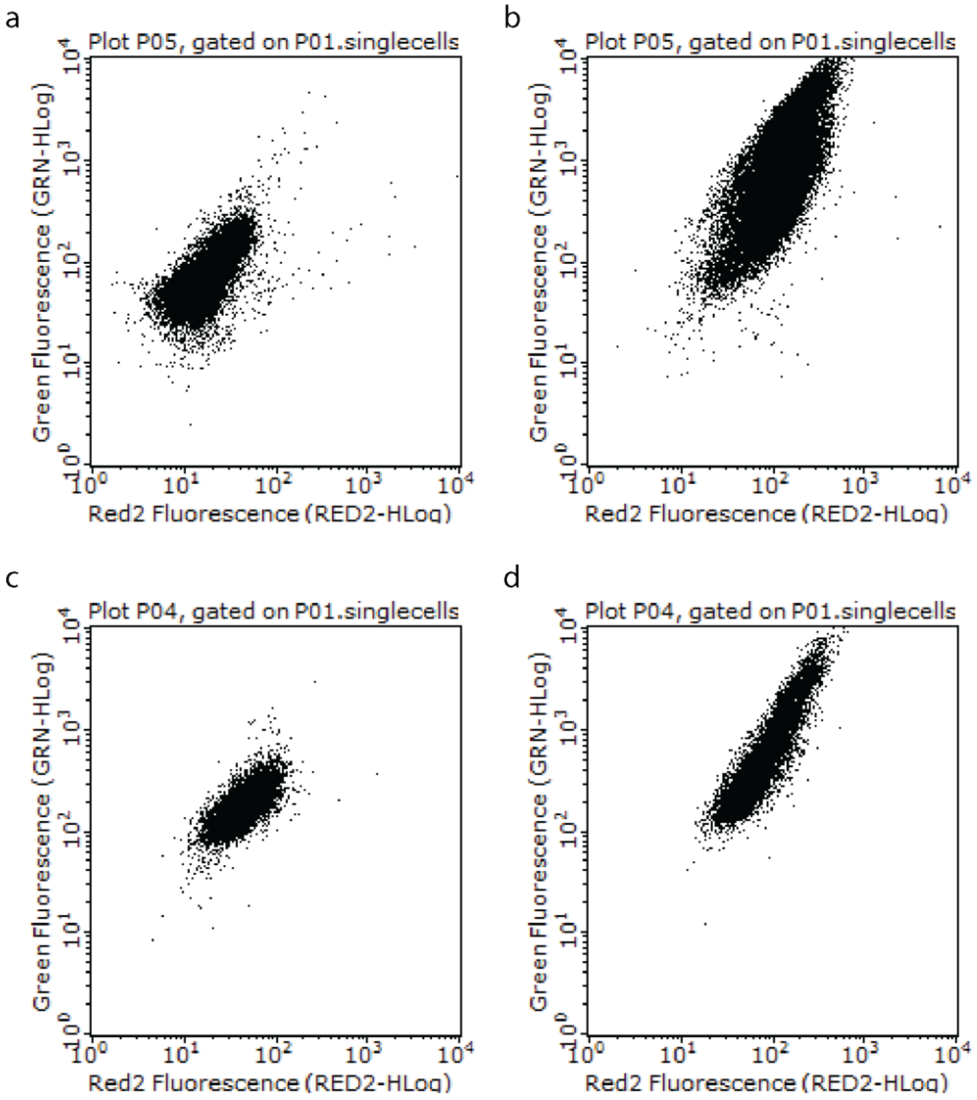

Supplementary Figure 8. Two-color immunoflow results show linear relationship between NLS-mCherry and Dll1 or EfnB2. (a) Naïve astrocytes stained with Dll1 antibody. (b) Dll1 lentivirus-infected astrocytes show linear relationship between mCherry fluorescence (Red2) and Dll1 488 antibody (Green). (c) and (d) show the same for naïve astrocytes and EfnB2-overexpressing astrocytes, each stained for EfnB2.

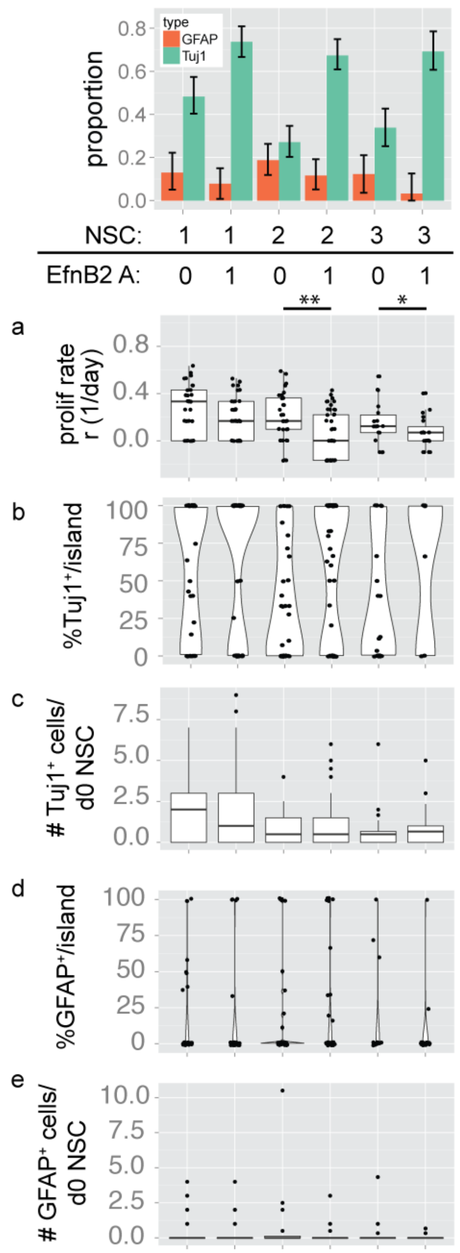

Supplementary Figure 9. Additional plots for NSCs patterned with EfnB2-expressing astrocytes. NSCs patterned with single hEfnB2-overexpressing cells exhibited enhanced Tuj1^{+} differentiation. (a) In the presence of an EfnB2-astrocyte, the mean proliferation rate decreases, which is statistical in the case of 2 initial NSCs (**, p=0.0012, t-test) and 3 initial NSCs (*, p=0.06, t-test). (b) NSCs exhibited enhanced Tuj1+ differentiation as evident in the wide distributions within the upper portion of the violin plots. (c) Boxplot of the number of Tuj1^{+} cells generated (initial NSC)$^{-1}$. No differences in the mean number of Tuj1+ cells were observed with the addition of one EfnB2 expressing astrocyte (for NSC = 1, 2, and 3). EfnB2 thus functions by increasing the fraction of NSCs islands^{-1} that give rise to a high proportion of Tuj1^{+} cells rather than increasing the number of Tuj1^{+} cells NSC^{-1} within a microisland. (d) Distribution of % GFAP^{+} island^{-1}. (e) Boxplot of the number of GFAP^{+} cells (initial NSC)$^{-1}$ reveal that very few GFAP^{+} cells are produced overall. All error bars are 95% confidence intervals.

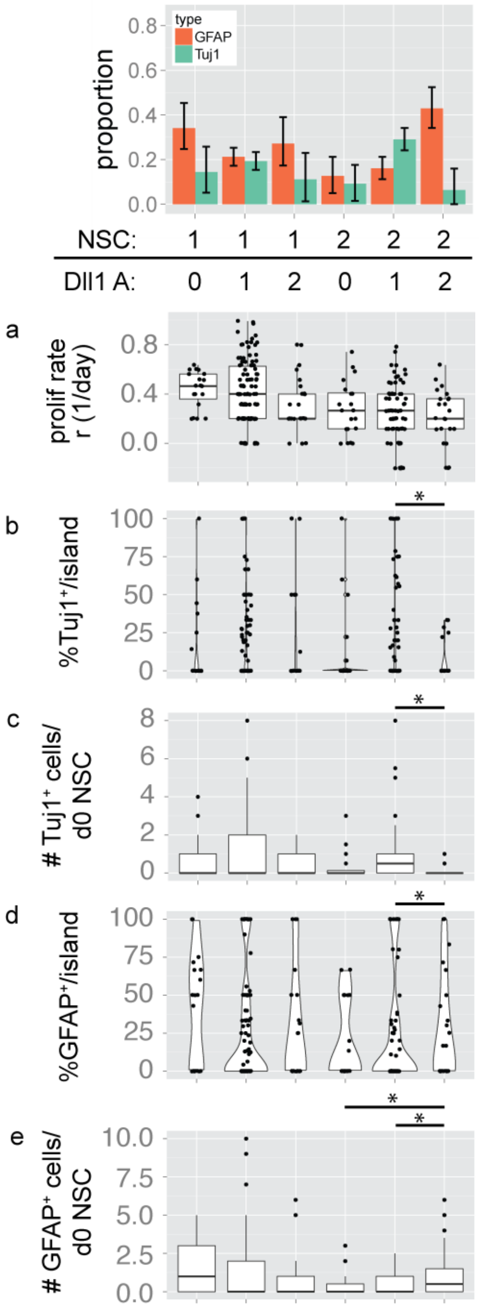

Supplementary Figure 10. Additional plots for NSCs patterned with Dll1-expressing astrocytes. NSCs patterned with single hDll1-overexpressing astrocyte exhibited reduced Tuj1. (a) Proliferation rates show no differences in the mean with the addition of Dll1-overexpressing astrocytes. (b) Narrow distributions for %Tuj1 island^{-1} reflect low Tuj1 differentiation rates. Asterisk indicates significant difference between samples. (c) Boxplot of the number of Tuj1^{+} cells generated (initial NSC)$^{-1}$. Asterisk indicates significant difference between samples. (d) Distribution of % GFAP island^{-1}. Asterisk indicates significant difference between samples. (e) Boxplot of the number of GFAP+ cells (initial NSC)$^{-1}$. Asterisk indicates significant difference between samples. All error bars are 95% confidence intervals; all p values obtained from ANOVA with Tukey HSD. *$p<0.05$.

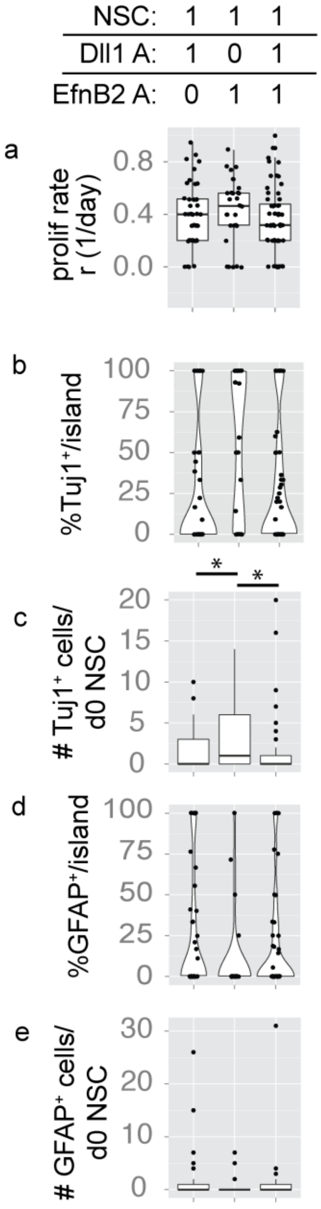

Supplementary Figure 11. Additional plots for NSCs patterned with EfnB2-expressing and Dll1-expressing astrocytes. (a) Proliferation rates show similar medians in patterns with only Dll1 and patterns with both signals. However, mean proliferation rates are not significantly different (p=0.6, ANOVA). (b) Violin plot showing similar distributions of %Tuj1 pattern^{-1} between patterns with only one Dll1-astrocyte and patterns with both types of astrocytes. By contrast, the %Tuj1 island^{-1} measurements with only one EfnB2 astrocyte were significantly elevated to higher levels (p<0.0.5, ANOVA with post-hoc Tukey Honest Significant Difference test). (c) Boxplot of the number of Tuj1^{+} cells with respect to initial NSC shows that islands with a Dll1-astrocyte and microislands with both a Dll1-astrocyte and a EfnB2-astrocyte produce significantly fewer Tuj1^{+} cells compared to microislands with only EfnB2 (p=0.022 and p=0.024 compared to the left and right columns, ANOVA with Tukey HSD). (d) Distribution of % GFAP island^{-1} does not reveal significant differences across samples (p=0.16, ANOVA). (e) Boxplot of the number of GFAP+ cells (initial NSC)$^{-1}$, with no significant differences across sample means (p=0.44, ANOVA). All error bars are 95% confidence intervals.

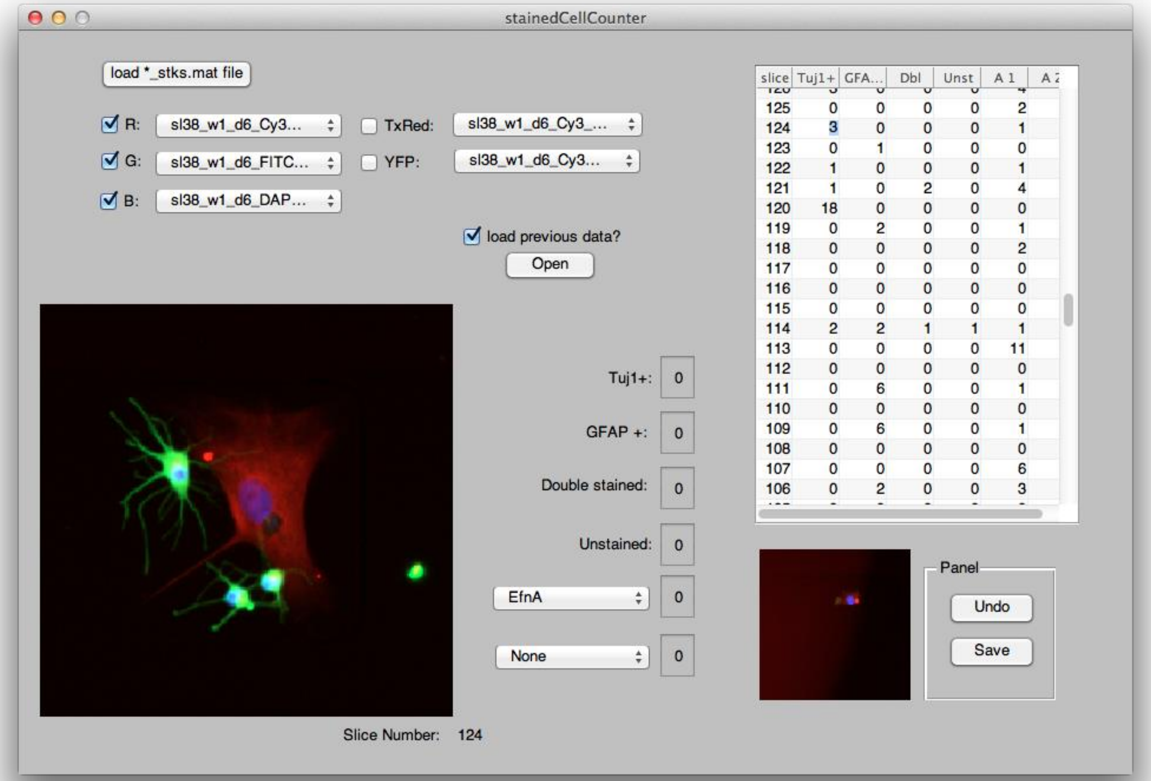

Supplementary Figure 12. Matlab GUI to facilitate the manual tabulation of cell counts. The software interface – described in greater detail in the Methods section – loads a series of images into red, green, and blue channels with optional extra channels for images of NLS-conjugated fluorescent proteins. Manually tabulated counts are entered into a table, which is stored in a data structure upon hitting 'save'. The editable table makes it easy to review and edit counts. To avoid bias, immunostaining counts are performed blinded to day 0 information on numbers and types of cells per island.

Supplementary Tables

#	community composition	n
1	1NSC + 1CortA	61
2	1NSC + 2CortA	59
3	2NSC + 1CortA	110
4	2NSC + 2CortA	67
5	2NSC + 3CortA	16
6	3NSC + 1CortA	22
7	3NSC + 2CortA	32
8	1NSC + 0EfnA	33
9	1NSC + 1EfnA	44
10	2NSC + 0EfnA	32
11	2NSC + 1EfnA	46
12	3NSC + 0EfnA	20
13	3NSC + 1EfnA	24
14	1NSC + 0DeltaA	19
15	1NSC + 1DeltaA	106
16	1NSC + 2DeltaA	21
17	2NSC + 0DeltaA	20
18	2NSC + 1DeltaA	65
19	2NSC + 2DeltaA	23
20	1NSC + 0EfnA + 1 DeltaA	45
21	1NSC + 1EfnA + 0 DeltaA	29
22	1NSC + 1EfnA + 1 DeltaA	57

Supplementary Table 1: Number of samples for each type of community composition.

Sequence Name	DNA Sequences (5’ – 3’)
A	GTA ACG ATC CAG CTG TCA CT TTTTTTTTTT TTTTTTTTTT TTTTTTTTTT TTTTTTTTTT TTTTTTTTTT TTTTTTTTTT ACTG ACTG ACTG ACTG ACTG
A’	GTA ACG ATC CAG CTG TCA CT TTTTTTTTTT TTTTTTTTTT TTTTTTTTTT TTTTTTTTTT TTTTTTTTTT TTTTTTTTTT CAGT CAGT CAGT CAGT CAGT
B	GTA ACG ATC CAG CTG TCA CT TTTTTTTTTT TTTTTTTTTT TTTTTTTTTT TTTTTTTTTT TTTTTTTTTT TTTTTTTTTT TCATACGACTCACTCTAGGG
B’	GTA ACG ATC CAG CTG TCA CT TTTTTTTTTT TTTTTTTTTT TTTTTTTTTT TTTTTTTTTT TTTTTTTTTT TTTTTTTTTT CCCTAGAGTGAGTCGTATGA
F	GTA ACG ATC CAG CTG TCA CT TTTTTTTTTT TTTTTTTTTT TTTTTTTTTT TTTTTTTTTT TTTTTTTTTT TTTTTTTTTT AGA AGA AGA ACG AAG AAG AA
F’	GTA ACG ATC CAG CTG TCA CT TTTTTTTTTT TTTTTTTTTT TTTTTTTTTT TTTTTTTTTT TTTTTTTTTT TTTTTTTTTT TTC TTC TTC GTT CTT CTT CT
G	GTA ACG ATC CAG CTG TCA CT TTTTTTTTTT TTTTTTTTTT TTTTTTTTTT TTTTTTTTTT TTTTTTTTTT TTTTTTTTTT AGC CAG AGA GAG AGA GAG AG
G’	GTA ACG ATC CAG CTG TCA CT TTTTTTTTTT TTTTTTTTTT TTTTTTTTTT TTTTTTTTTT TTTTTTTTTT TTTTTTTTTT CTC TCT CTC TCT CTC TGG CT
CoAnchor	AGT GAC AGC TGG ATC GTT AC

Supplementary Table 2: Complementary pairs of cell-labelling and surface-printed DNA sequences in addition to the CoAnchor sequence for stabilizing cell-labelling oligonucleotides.

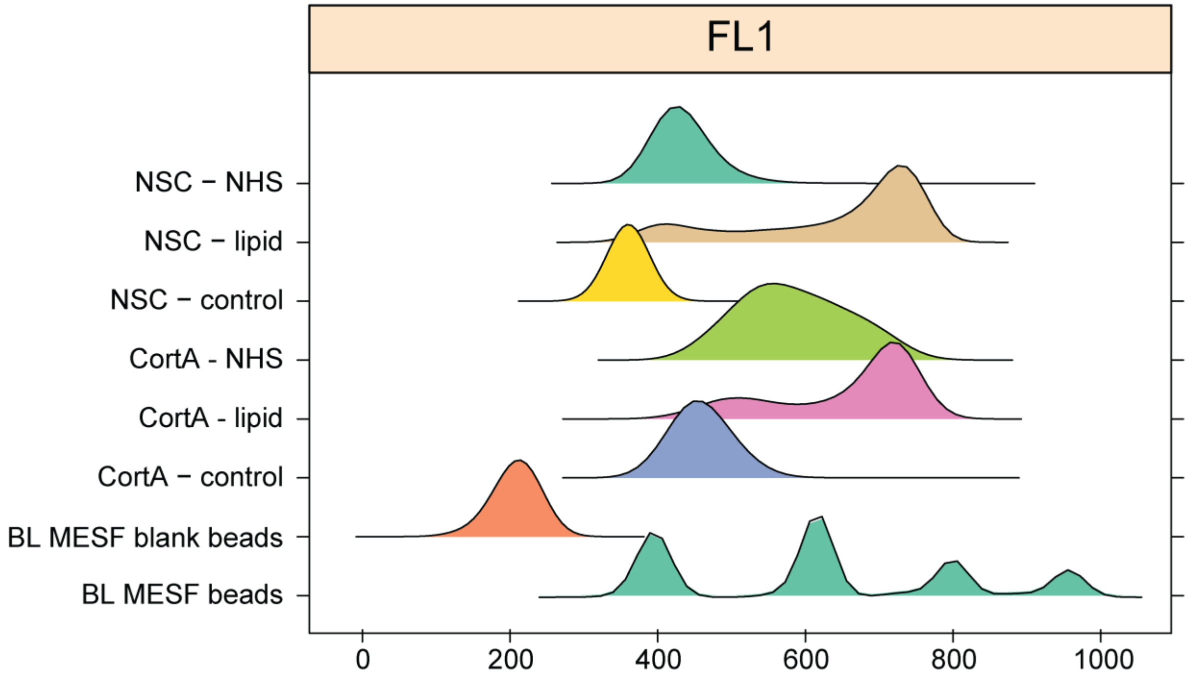

Type	Peak (strands cell^{-1})	5th percentile (strands cell^{-1})	95th percentile (strands cell^{-1})
NSC - NHS	4,606	3,125	10,727
NSC – lipid	107,809	3,701	139,626
NSC – control	2,437	1,808	3,556
CortA – NHS	18,355	7,498	84,914
CortA - lipid	90,136	7,498	135,521
CortA - control	5,732	3,666	13,619

Supplementary Table 3: Assessment of DNA incorporation into cells. After cell labeling by lipid DNA or NHS-DNA, cells were incubated with Alexa 488-conjugated complementary oligonucleotides and assessed by flow cytometry. Lipid DNA outperformed NHS-DNA for both NSCs (108k vs. 5k strands cell^{-1}) and astrocytes (90k vs. 18k strands cell^{-1}).

Supplementary Notes

Supplementary Note 1:

Our ability to measure many metrics from each patterned community yields data that can be displayed in a variety of ways. Here, we provide extensive descriptions of the supplementary plots from each experiment (below) with some guidelines for interpretation.

Proliferation rate: The proliferation rate is calculated as described in the Methods section. In these plots, a boxplot of the proliferation rate measurement is overlaid with jittered points, each representing a measurement from one island.

% Tuj1$^+$ island^{-1}: For each island, the %Tuj1$^+$ island^{-1} value is calculated by dividing the number of Tuj1$^+$ cells over total number of NSC-progeny at day 6. For each type of community composition, a violin plot of the %Tuj1$^+$ island^{-1} distribution is overlaid with jittered points, each representing a measurement from one island. For most plots there may be a large number of 0% Tuj1$^+$ measurements, making it impossible to infer an idea of the distribution from looking at the jittered points alone. Thus, the interpolated lines of the violin plot provide a better sense of the true distribution of values along the 0-100% y-axis.

# Tuj1$^+$ cells (d0 NSC)$^{-1}$: For each microisland, the # Tuj1$^+$ cell at day 6 is divided by the total number of NSCs at day 0. This provides a measure of the average number of Tuj1$^+$ cells produced from each NSC. In communities with only one initial NSC, this is a measure of the absolute number of Tuj1$^+$ cells produced. These data are represented as a boxplot with the line designating the median, with upper and lower "hinges" corresponding to the first and third quartiles (the 25th and 75th percentiles).

% GFAP$^+$ island^{-1}: Similar to % Tuj1$^+$ island^{-1}. In general, since GFAP marker expression is less frequent than Tuj1 expression, we have less statistical power when analyzing GFAP data. Note that the GFAP+ NSC progeny can readily be distinguished from the cortical astrocytes (which are much larger and flatter) and, in many cases, by cortical astrocyte expression of a NLS fluorescent protein.

# GFAP$^+$ cells NSC^{-1}: Similar to # Tuj1$^+$ (d0 NSC)$^{-1}$

For each of these plots, interesting and significant results are described in the figure legend. Plots without significant differences are presented without comment. For ANOVA, we subset the data based on the number of initial NSCs to assess the impact of additional astrocytes.
